# Smoking Behavior among Coronary Heart Disease Patients in Jordan: A Model from a Developing Country

**DOI:** 10.3390/ijerph7030751

**Published:** 2010-02-26

**Authors:** Nesrin N Abu-Baker, Linda Haddad, Omar Mayyas

**Affiliations:** 1 School of Nursing, Jordan University of Science & Technology, P.O Box 3030, Irbid 22110, Jordan; E-Mail: nesrin@just.edu.jo; 2 Department of Family and Community Health, Institute for Drug and Alcohol Studies, School of Nursing, Virginia Commonwealth University, Richmond, P.O. Box 980567, VA 23298-0567, USA; 3 Nursing Department, Technical Institute for health training, Jeddah 21361, Saudi Arabia; E-Mail: onmayyas05@nurs.jutd.edu.jo

**Keywords:** smoking cessation, coronary heart disease, quitter smoker, persistent smoker, Jordan

## Abstract

The purpose of this study was to compare the frequency of cigarette smoking before and after diagnosis of Coronary Heart Disease (CHD), detect the reasons that discourage quitting smoking and resources of advice about quitting, and investigate the relationship between smoking behaviors and demographic variables. A convenient sample of 300 CHD patients from cardiac outpatient clinics participated. Before disease occurrence, nonsmokers composed 40% of all participants, former smokers 11.7%, and current smokers 48.3%. Surprisingly, after disease occurrence only 29.7% of the patients quit smoking, while 60.7% continued smoking, and 9.6% relapsed. The most frequent reasons given by smokers for not quitting smoking were “do not incline to stop smoking” (25.6%) and “craving for a cigarette” (25%). Doctors were cited most frequently as the reason individuals quit smoking (19.0%). The Jordanian health care system needs to implement systematic intensive smoking cessation programs to maintain and promote CHD patients’ motivation to quit smoking.

## 1. Introduction

Coronary heart diseases are emerging as a major health problem in the Eastern Mediterranean region, where the proportion of deaths from CHD ranges from 25% to 45% [1]. More specifically, CHD accounted for 35% of the overall mortality in Jordan [1,2]. As a developing country, Jordan has a high rate of smoking prevalence compared to developed countries [1–3]. Furthermore, Jordanians view smoking as a social habit; having coffee and cigarettes with friends and family members is deeply rooted in the culture. During 2002−2006, 50% of adult men and 11% of adult women smoked [4–6]. A recent series of surveys in Jordan estimated smoking rates of 24.9% among youth, with a 76.3% self-report of SHS exposure [6], 22.4% among male physicians [7], and 28.6% among college students [8,9], which rose to 35% in 2008 [10]. Since cigarette smokers are two-to-three times more likely to die from coronary heart disease than nonsmokers [2], this poses immense problems for reducing CHD in Jordan. Thus, all evidence indicates that a country with high smoking prevalence faces major risks of CHD related morbidity and mortality among the population [1–5].

Furthermore, due to the impact of CHD on public health and the escalating cost of health care, strategies for smoking cessation are becoming increasingly important for Jordan. Also, the beneficial effect of smoking cessation on prognosis after the occurrence of CHD due to a decreased risk of secondary cardiovascular disease events is very obvious [11–13].

### 1.1. Factors That Discourage CHD Patient from Quitting Smoking

Researchers have found that 29% of cardiac patients in Western countries who were smokers have persisted in their smoking behaviors or relapsed after hospital discharge [14]. There is an accumulation of evidence that several factors are related to quitting tobacco smoking: socioeconomic status [15]; Nicotine dependence as measured by number of cigarettes smoked per day and years of smoking [16]; gender [17]; genetics factors [18]; and health risk behaviors, psychological variables including health concerns, and environmental external variables related to antismoking climate in a population [19,20].

Offering smoking cessation counseling to all hospitalized smokers is effective as long as supportive contacts continue for more than one month after discharge. Adding nicotine replacement therapy to counseling may further increase smoking cessation rates and should be offered when clinically indicated, especially to hospitalized smokers with nicotine withdrawal symptoms [21].

Furthermore, systematic review finds that smoking cessation counseling that begins during hospitalization and provides supportive contacts for over one month after discharge increases the odds of smoking cessation at 6 to 12 months by 65% over what is achieved by hospitalization alone. There is no evidence that less intensive counseling interventions, particularly those that do not continue after hospital discharge, are effective in promoting smoking cessation [2,22]. The intensive counseling intervention is effective when provided to all hospitalized smokers, regardless of admitting diagnosis [22,23].

Smoking cessation is more effective than any other medical regimen or modality for secondary prevention of CHD [24]. Smoking cessation decreases mortality by 36% [25], nonfatal reinfarction by 32%, and repeated coronary artery bypass graft rates over 50% [23]. In addition, smoking cessation is cost-effective compared to other interventions [23].

The increase in knowledge about efficacious ways to help CHD smokers quit has not been translated into effective clinical practice in Jordan. Developing, implementing, and evaluating smoking cessation interventions for CHD Jordanian patients have to start with a systematic assessment and overview of the target population’s smoking behavior and cessation practice. Furthermore, no studies about the smoking behavior and cessation among CHD patients in Jordan were found. Therefore, the purpose of this study was to obtain an overview of smoking status before and after the diagnosis of CHD, to look at the reasons that discourage coronary heart disease patients from quitting smoking after the diagnosis, to detect the resources of advice regarding quitting smoking, and to investigate the relationship between demographic variables (age, gender, educational level, and income) and the prevalence of cigarette smoking among current smokers after the diagnosis of their disease.

## 2. Methodology

### 2.1. Design

A descriptive cross-sectional design was used to answer the study research questions.

### 2.2. Setting and Sample

The study was carried out in the city of Irbid, in northern Jordan. Irbid is the second largest city in the country [3]. The population of this study consists of all CHD patients of Irbid governorate who are referred to cardiac outpatient clinics as being diagnosed with one of the coronary heart diseases: stable and unstable angina, myocardial infarction (MI), atherosclerosis, cardiomyopathy, endocarditis, valvular heart disease and patient’s post percutaneous transluminal coronary angioplasty (PTCA), acute coronary syndrome (ACS), and coronary arterial bypass graft (CABG). Irbid governorate has only two large public cardiac outpatient clinics, one in Princess Basma Education Hospital and the other in King Abdullah University Hospital. Usually 80% [1] of CHD in Irbid visit those two outpatient clinics while the other 20% of the patients visit 15 private sector clinics across the governorate [3].

The study sample consisted of 300 patients from the outpatient clinics of King Abdullah University Hospital (679 beds) and Princess Basma Educational Hospital (300 beds) [3]. A convenient sample was taken from each clinic. The sample size was calculated using the analysis program “Epicalc 2000, version 1.02”, which assumes that the expected percentage of cigarette smoking is 45%, given a level of significance of 0.05 and a 95% confidence interval among CHD patients.

The inclusion criteria included patients who are younger than 80 or older than 29 years of age at the time of CHD onset and have been diagnosed with CHD for 3 months or more. The reason for the wide margin of age criteria is due to the fact that incidence and prevalence of CHD is around 40%, and the main cause of death in Jordan is related to CHD [2]. However, patients who had debilitating illnesses that prevented the completion of formal interviews were excluded from the study.

### 2.3. Measurement

A cigarette smoking questionnaire was used to investigate cigarette smoking behavior among coronary heart disease patients. The questionnaire consisted of three major components: demographic data, disease history, and smoking history. Demographic data included age, gender, height, weight, marital status, level of education, and household income. Smoking history included, smoking status, whether or not the patient smoked before or after the disease, length of smoking period and number of cigarettes smoked before and after the disease, length of quitting period after diagnosis, sources of advice to quit smoking, and reasons that discourage the patient from quitting. Medical records were used only to obtain the disease history data, which included type of disease diagnosis, date and length of diagnosis, and history of other diseases.

The questionnaire was developed by the researcher and based on previous literature. To ensure content validity, the questionnaire was evaluated by a panel of experts who were selected based on their qualifications and experience in nursing research and education in Jordan.

### 2.4. Pilot Study

The instruments were pilot tested to identify ambiguities in questions, times required for completing the questionnaire, the adequacy of the data collection plan, and any difficulties that might be encountered by the participants in reading or understanding the questionnaire. 30 participants from general medicine clinics participated in the pilot study. Results of the pilot study showed that the questionnaire was clear, easy to read, and required around 20 minutes to be completed. The internal consistency reliability of the instrument and coefficients were computed. The reliability coefficient of the questionnaire was 0.70.

### 2.5. Human Participation Protection

Formal permission to conduct the study in the hospitals was obtained from the directors of the two hospitals. Approvals were also obtained from the Institutional Review Board (IRB), the ethical Committee at Ministry of Health for Princess Basma Educational Hospital, and from Research/Ethics Committee at King Abdullah University Hospital.

Ethical codes were addressed in the cover letter of the questionnaire. The researcher explained the purpose of the research to all participants. Participants were asked for their permission to access some information from their medical records. At the same time an informed consent was obtained from each participant. The assurance of anonymity was addressed prior to request for participation. Data collection methods were designed to protect the confidentiality of the information obtained by assigning a code number to all data collection methods. The code number was helpful in reviewing the medical record. Any link between code numbers and patient names was destroyed. Only aggregated data were reported. Furthermore, participants were assured that their participation in the study was voluntary and that they could withdraw at any time.

### 2.6. Data Collection Procedures

Every patient who agreed to participate was recruited and interviewed for 15−20 minutes in the outpatient clinics. Self-reported data were collected using a structured interview to avoid missing data and to assist participants in understanding research questions better. The questionnaire used in this study had a structured format for asking all participants the same questions. Researchers asked participants for access to some information from the patients’ medical records. After the interview was completed, specific data were obtained from patients’ medical records to verify the medical diagnosis. Data collection took about three months (20 May to 24 July 2007) to be completed; the response rate was 98%. Data collection was conducted by the same researchers across the two hospitals. Data collection was carried out in the same manner for all participants.

### 2.7. Data Analysis

Data were analyzed using the statistical package for social science (SPSS) computer program version 15. Descriptive statistics were performed to describe all variables. Chi-square was used to investigate the association between the study variables. T-test was used to study the differences between the mean of cigarettes smoked before and after disease occurrence. The significant of the association was tested at an alpha level of ≤0.05.

## 3. Results

### 3.1. Sample Characteristics

A total of 300 patients with coronary heart disease were interviewed in the two hospitals. The sample included 100 patients from the medical outpatient clinics in Princess Basma Educational Hospital and 200 patients from the cardiac outpatient clinics in King Abdullah University Hospital.

The mean age was 56.43 years (SD = 11.65). The participants who were between 60 and 69 years of age had the maximum percentage, 28.3% (n = 85), while the age group between 30 and 39 years of age had the minimum percentage, 8.7% (n = 26). Male patients represented 77.7% (n = 233) of the participants, while female patients represented 22.3% (n = 67). Moreover, 62.0% (n = 186) of the participants had less than a high school education, 16.3% (n = 49) completed high school, 9.3% (n = 28) had an associate degree, and 12.3% (n = 37) had a university degree. About 60.0% (n = 180) of the sample had an annual income equal to or less than $3,600, while 40.0% (n = 120) had an annual income more than $3,600, which is less than the average per capita income of $5,000 [26].

### 3.2. Smoking Behavior before the CHD Incidence

Current smokers are defined as persons who reported that they smoked at the time of CHD incidence. Data analysis revealed that current smokers composed 48.3% (n = 145) of all participants. Of those current smokers, 89% (n =129) were men, and 11% (n = 16) were women. Former smokers, on the other hand, are persons who reported that they had smoked but had quit before the incidence of CHD. Former smokers composed 11.7% (n = 35) of all participants.

Nonsmokers are defined as persons with no history of cigarette smoking based on self-report. One hundred and twenty participants did not smoke before CHD occurrence, but there was one participant who started to smoke after the disease occurrence. Therefore, Nonsmokers, comprised 40.0% (n = 120) of all participants (see Table 1).

Furthermore, the mean number of cigarettes per day before CHD occurrence was 33.4 (SD = 17.67), and this mean decreased to reach 26.74 (SD = 19.82) after CHD occurrence (see Table 2). There was a significant difference in the number of cigarettes smoked before CHD occurrence, t (101) = 19.11 P < 0.01, and the diagnosis of CHD occurrence, t (101) = 13.62, P < 0.01.

### 3.3. Smoking Behavior after the CHD Incidence

Quitter smokers are defined as persons who reported that they were smoking at the time of the incidence of CHD and quit after the disease occurrence for three months or more. There were 33 participants who quit smoking directly after CHD occurrence, and 10 participants quit at the time of interview for 3 months or more. So quitter smokers, as statistical analysis revealed, comprised 29.7% (n = 33 +10 = 43) of current smokers.

Persistent smokers are defined as persons who reported that they were smoking at the time of the CHD incidence and continued to smoke or relapsed during follow-up. Data analysis revealed that 88 participants continued smoking at the time of disease occurrence and then persisted after disease occurrence. According to the operational definition of a quitter smoker, if the quitting period was less than 3 months, the participant was considered as a persistent smoker (n = 14). So persistent smokers, as statistical analysis revealed, comprised 70.3% (n = 88 + 14 = 102) of current smokers (see Table 1).

### 3.4. Reasons that Discourage CHD Patients from Quitting Cigarette Smoking after the Diagnosis of Their Disease

One hundred and sixty eight participants identified the reasons that prevent smokers from quitting smoking after disease occurrence. Data analysis revealed that the most frequent reasons were “do not incline to stop smoking”, 25.6% (n = 43), “craving for a cigarette”, 25.0% (n = 42), “other people around me smoke”, 11.3% (n = 19), and “loss of a way to handle stress”, 10.7% (n = 18). The least frequent reasons were “cost of medicines or products that help to quit”, 2.4% (n = 4) and, finally, “availability of cigarettes at a low cost”, 0.6% (n = 1) (see Table 3).

### 3.5. Resources of Advice Regarding Quitting Smoking

Two hundred and twenty six participants identified the resources of advice given to CHD patients about how to quit smoking. Statistical analysis revealed that the most frequent resource was “doctor”, 19.0% (n = 43), then “family or relative”, 12.8% (n = 29), and then “nurse”, 10.6% (n = 24). The least frequent resource was “magazine, book, or newspaper”, 0.9% (n = 2). The percentage of participants who answered “no one” was 17.7% (n = 40) (see Table 4).

### 3.6. Demographic Variables and Smoking Behavior Relationship

Data analysis revealed that there was a statistically significant association between smoking behavior after CHD occurrence and age group, X^2^ (df = 4, N = 145) = 11.15, p = 0.03 (Two tailed), and income level, X^2^ (df = 1, N = 145) = 5.6, p = 0.02 (Two tailed). The age groups of 40−49 and 50−59 had the highest percentages of persistent smokers (74.3% and 87.5% respectively) compared to quitter smokers. The patients with annual incomes of more than $3,600 had a higher percentage of persistent smokers, 81.8% compared to quitter smokers (see Table 5). However, there was no statistically significant association between smoking behavior and gender, X^2^ (df = 1, N = 145) = 1.03, p = 0.31 (Two tailed), or educational level, X^2^ (df = 3, N = 145) = 1.99, p = 0.58 (Two tailed).

## 4. Discussion

The researchers wanted to compare this study’s findings with other studies of Jordanian patients with CHD, but none could be found. However, compared to the last national survey [3], smoking prevalence among male and female CHD patients was higher than prevalence of smoking among the general adult population (male and female) of Jordan. In Comparison to other similar Western studies, Rallidis, Hamodraka, Foulidis, & Pavlakis [27] followed myocardial infarction patients after 6 months of the diagnoses; their findings revealed that 60% of patients were smokers at the time of MI, while at interview 32.5% were persistent smokers, 33.5% were former smokers, and 34% never smoked. Another Japanese study by Kinjo [20] followed myocardial infarction patients after 3 months of the diagnosis and continued to follow them for five years. Their study revealed that 31.9% were nonsmokers, 12.9% were former smokers, and 55.2% were current smokers. Of the current smokers, 74.2% had quit smoking, and 25.8% were persistent smokers at three months after hospital discharge. In conclusion, the percentage of the persistent smokers in the current study is much higher than the percentages of these two studies.

Furthermore, there was a higher proportion of persistent smokers in the current study than in the study that was undertaken in 15 European countries during 1999–2000, where persistent smokers comprised 21% of the study sample after CHD occurrence [28]. This difference might be explained by the differences in cultural attitudes toward smoking and cessation resources between Western developed countries and developing low to middle income countries such as Jordan. Jordanian society encourages cigarette smoking, especially among men. CHD patients believe that smoking cessation will not prolong their lives. Furthermore, Jordan’s health institutes lack any systematic, comprehensive smoking cessation programs. Ironically, Jordan is one of the first Middle Eastern countries to pass legislation banning smoking in all public places, including hospitals, and encourage the development of systematic smoking cessation programs. Unfortunately, such bylaws have not been implemented and enforced. Smoking is still socially acceptable in Jordan, where citizens will frequently ask, “How come you do not smoke?” Furthermore, it is quite common to walk into any private or public medical building and see patients, clients, and the medical team smoking in the waiting area.

It should be noted that participants who smoked after CHD occurrence reported that the most frequent reasons for not quitting were “do not incline to stop smoking” (25.6%) and “craving for a cigarette” (25.0%). This is mainly explained by the nicotine dependence among smokers, evidenced by their high daily cigarette consumption that makes addiction an important issue to address in any cessation program in Jordan. Such a conclusion implies that nicotine dependence has a high predictability of smoking cessation among CHD patients. This finding was also consistent with the Atsuhiko, Yoshio, Hiroshi, & Norito study [29], which focused on the predictability of two alternative questionnaires of nicotine dependence among patients with CHD. In this study, patients with a high Tobacco Dependence Screener score were significantly less likely to quit smoking than the group with a low score. Unfortunately, tobacco dependence treatment is not a common practice in Jordan. In fact, most of the nicotine pharmacological treatments do not even exist in Jordan.

The results showed that the he first two reasons for not quitting were “loss of a way to handle stress” and “have a history of depressive symptoms.” These reasons can be explained by the fact that cigarette smoking has definite immediate positive effects; it relieves minor depression, helps suppress little fits of anger, enhances concentration and short-term memory, and produces a modest sense of well being [15]. Thus, it can be concluded that nicotine dependence discourages participants from quitting smoking more than economic pressures.

In regard to the source of advice regarding quitting, many studies revealed that a health care provider’s advice can successfully help patients to quit smoking [30,31]. Our study found that among all health care providers, doctors had the highest reported percentage of effectively advising participants to stop smoking. This finding could be related to Jordanian culture, in which doctors are perceived to be the most trustworthy health care providers and, therefore, the most frequent source of advice. “Nurses” formed a source of advice for about 10.6% of participants. This is considered a low percentage compared to other sources, which implies that nurses need to improve their role in health promotion programs for smoking cessation, especially since literature indicates that primary care nurses can effectively provide recommended smoking-cessation counseling [31]. It is worth noting that a recent Jordanian study [32] revealed that Jordanian health professionals received insufficient training related to tobacco use and control. Moreover, Jordanian medical and nursing schools inadequately teach tobacco intervention skills. There is a lack of integration of tobacco dependency information throughout Jordan’s medical and nursing curricula [7].

Moreover, mass media, magazines, and books or newspapers had low percentages as sources of advice, indicating that little health education is gained from them. One explanation is the limited and scarce organized mass media campaigns about smoking and health in Jordan. However, family or relative and friend had also a high percentage as a source of advice. A possible explanation for the family and relative’s high effect is that Arabs are highly affiliative and value the family unit over the individual. It would therefore appear that this might be a reflection of the culture and its norms, as individual identity derives from family and commitment to family affairs. This implies that any smoking cessation program in Jordan needs to address the family as part of the cessation counseling.

Furthermore, the findings revealed that around 18% of the participants have not received any kind of advice to quit smoking at all, which is an alarming finding that could be explained by the fact that hospitals in Jordan do not provide any advice on cessation to the patients. Knowing that hospitals are a key channel for delivering smoking cessation interventions to the patients, one can conclude that public hospitals in Jordan are losing opportunities to motivate patients to quit smoking. Also, another explanation could be related to the fact that clinicians in Jordan do not inquire about tobacco usage. So they may even not know that their patients are smokers.

The results of the current study showed that there is a statistically significant association between age and smoking behavior after CHD occurrence. In general, Persistent smokers were younger than quitter smokers, which may indicate that older participants were more aware of their health than younger patients. This proportion is consistent with the study that was conducted in Piraeus, which also found that persistent smokers were younger than nonsmokers [28].

It appears that the pattern between income and smoking behavior after CHD occurrence is different from those reported in developed countries. There was statistically significant association between income and smoking behavior after CHD occurrence. The population with a higher annual income had a higher percentage of persistent smokers compared to quitter smokers. In contrast, a study aimed at examining the association of income with CHD risk factors by gender and smoking status was conducted in Norway. Here, the results revealed no decrease in smoking prevalence by increasing income level among women, whereas there was a decrease in prevalence among male current smokers as income levels increased (P ≤ 0.05). Modest increases in the prevalence of former smokers were noted for both sexes as income levels increased [16].

### 4.1. Study Limitations

The findings of the current study should be interpreted while taking into consideration the convenient sampling approach that limits the generalizability of the findings and the period of answering the questionnaire that took place at the time of referral to the doctor in the waiting area, which might have provided a chance for unsatisfactory answers and lead to some inaccuracy. The data used to asses abstinence and relapse are self-reported, and it is possible that participants may have misreported their smoking status. Another limitation of this study was the use of a cross-sectional study design rather than a longitudinal or clinical trial; therefore, causal associations cannot be assumed. Also, there is a greater likelihood that patients who are heavier smokers may not have been in the sample because of smoking related deaths. Finally, the definition of smoker in our study neither considered nor included the waterpipe/hookah smokers who are highly prevalent in Jordan. This may have led to patients mislabeling themselves as nonsmokers because they only smoked tobacco in a waterpipe/hookah.

### 4.2. Recommendations

This study’s results provide important baseline information about frequency of smoking before and after diagnosis of (CHD) and detect the reasons that discourage patients from quitting smoking and the resources available to patients that might help them quit smoking. Cessation programs among CHD patients should be introduced in both medical and nursing curricula in Jordan. The role of mass media campaigns in health promotion programs in general and smoking cessation programs in particular should be enhanced. Also, strategies and enforceable policies that maintain hospitals and medical centers as non-smoking zones are needed to further encourage a smoke free society in Jordan.

### 4.3. Practice Implications

There are a variety of factors that contribute to why Jordanian patients are unable to quit smoking. Health care providers need to deliver a clear and strong message to every CHD patient. Health care providers can raise patients’ motivation to quit smoking by emphasizing that smoking is a major cause of their heart disease and that stopping smoking can reduce morbidity and mortality [19]. Intensive follow-up contact by telephone or appointment and support from health care providers after discharge might be helpful to maintain patients’ motivation to remain abstinent. Discussing smoking cessation programs with patients should involve family members. Health care professionals need to express their understanding to family members who can attempt to persuade patients to stop smoking and encourage family members to use more positive support behaviors to help patients [7,32]. In other words, implementations of systematic strategies that institutionalize cessation services are needed; these may include training health care providers to deliver effective treatments, including tobacco dependence pharmacological treatment.

### 4.4. Conclusion

Despite the study limitations, this study presents several important findings. This is the first study to investigate smoking behavior among CHD patients in Jordan. The majority of the CHD patients continue to smoke after their disease occurrence, suggesting that these patients need strong, systematic smoking-cessation programs. A high proportion of these patients were nicotine addicted, therefore any smoking cessation program has to include intervention for the nicotine addiction. Furthermore, there is a need to improve intensive follow-up contact and support from health care providers after hospital discharge.

## Figures and Tables

**Table 1.** Smoking behavior before and after CHD occurrence (n = 300).

**Smoking Status Before CHD occurrence**	**Frequency**	**Percentage**
Current Smokers	145	48.3
Former Smokers	35	11.7
Nonsmokers	120	40.0
**Total**	300	100.0
**After CHD occurrence**		
Persistent	88	60.7
Relapsed	14	9.6
Quitter	43	29.7
**Total**	145	100.0

**Table 2.** Number of cigarettes smoked and period of smoking before and after CHD occurrence.

**Period of smoking and number of cigarettes**	**N**	**Minimum**	**Maximum**	**M (SD)**
Period of smoking before CHD occurrence in months	102	12	660	328.1 (153.5
Period of smoking before CHD occurrence in years	102	1	55	27.3 (12.8)
Number of cigarettes per day before CHD occurrence	102	3	100	33.4 (17.6)
Period of smoking after CHD occurrence in months	102	2	384	48.6 (65.0
Number of cigarettes of per day after CHD occurrence.	102	1	100	23.7(19.8)

**Table 3.** Reasons that discourage CHD patients from quitting smoking.

**Reasons**	**Frequency**	**Percentage**
Do not incline to stop smoking	43	25.6
Craving for a cigarette	42	25.0
Other people around me smoke	19	11.3
Loss of a way to handle stress	18	10.7
Have a history of depressive symptoms	12	7.1
Lack of support from others to quit	10	6.0
Fear of gaining weight	7	4.1
No or few classes that help me to quit are available in the health centers and hospitals	6	3.6
Some other reasons	6	3.6
Cost of medicines or products that help quit	4	2.4
Availability of cigarettes at a low cost	1	0.6
**Total**	168	100.0

**Table 4.** Resources of advice to stop smoking.

**Resources of advice**	**Frequency**	**Percentage**
Doctor	43	19.0
No one	40	17.7
Family or relative	29	12.8
Nurse	24	10.6
Mass media	8	3.5
Friend	6	2.7
Other	4	1.8
Magazine, book or newspaper	2	0.9
All of listed	70	31.0
**Total**	226	100.0

**Table 5.** Smoking behavior after CHD occurrence by age and income category.

Age category*	**Smoking status after CHD disease occurrence**		**Total**
	**Persistent n (%)**	**Quitter n (%)**	
30–39	11 (57.9)	8 (42.1)	19 (100)
40–49	26 (74.3)	9 (25.7)	35 (100)
50–59	35 (87.5)	5 (12.5)	40(100)
60–69	24 (61.5)	15 (38.5)	39 (100)
70–79	6 (50.0)	6 (50.0)	12 (100)
**Total**	102 (70.3)	43 (29.7)	145 (100)
Income category **			
≤ 3600$	57 (63.3)	33 (36.7)	90 (100)
>3600$	45 (81.8)	10 (18.2)	55 (100)
**Total**	102 (70.3)	43 (29.7)	145 (100)

* X^2^ = 11.15, df = 4, *p* = 0.03 (Two tailed).

** X^2^ = 5.6, df = 1, *p* = 0.02 (Two tailed).
